# Community satisfaction with the urban health extension service in South Ethiopia and associated factors

**DOI:** 10.1186/s12913-015-0821-4

**Published:** 2015-04-16

**Authors:** Ephrem Lejore Sibamo, Tezera Moshago Berheto

**Affiliations:** Hadiya Zone Health Department, Health extension program Coordinator, P.O.BOX 29, Hosanna, Ethiopia; Lecturer at Schoolof Public Health, College of Health Sciences, Wolaita Sodo University, P.O.Box138, Sodo, Ethiopia

**Keywords:** Urban Health Extension Program, Urban Health Extension Professionals, Satisfaction, Hadiya Zone, Ethiopia

## Abstract

**Background:**

The urban health extension program (UHEP) is an innovative government plan to ensure health equity by creating demand for essential health services through the provision of appropriate health information at a household level. It aims to improve the overall health of a community through active participation and utilization of services, which depends on satisfaction and acceptance of the program. However, there is no study on community satisfaction with the services provided by the UHEP in Ethiopia. This study was aimed to assess the level of community satisfaction with the UHEP in Hadiya Zone, South Ethiopia, and the associated factors with it.

**Methods:**

A community based cross-sectional study, using quantitative and qualitative methods of data collection, was employed. Quantitative data were collected from 407 respondents using a structured questionnaire. Descriptive statistics, bivariate and multiple regression analyses were performed. P-value less than 0.05 and 95% confidence intervals were used to determine an association between independent and dependent variables. Qualitative data were collected through focus group discussions with village health committee members and model families in Hadiya Zone.

**Results:**

The majority (67.4%) of respondents were satisfied with the services provided by the UHEP. The communities’ perceptions of technical competency (ß = 0.425; 95% CIs 0.084, 0.34), interpersonal relationships (ß = 0.506; 95% CIs 0.216, 0.797), and perceived accessibility of services (ß = 0.752; 95% CIs 0.064, 0.86) were independent predictors of satisfaction (P < 0.05). In addition, the marital status, knowledge, and attitudes of the respondents were associated with community satisfaction.

**Conclusion:**

A community’s satisfaction with UHEP has supposed to have a significant influence on the community’s utilization of the services and implementation of the program. The present study have clearly shown that majority of the respondents were satisfied with the services provided by urban health extension program.

## Background

A Health Extension Program (HEP) is “a package of basic and essential promotive, preventive, and curative health services targeting households in a community based on the principle of Primary Health Care (PHC) to improve families’ health” [1]. A HEP was initiated in 2003 in rural communities in Ethiopia as part of the health sector development program, by expanding physical health infrastructure and training and deploying a cadre of female Health Extension Workers (HEWs) [2].

The main objectives of HEP are to improve equity and access to essential health interventions at the community level by ensuring ownership and participation of the community, increasing health awareness and skills among community members, improving utilization of PHC services and promoting life styles which are conducive to good health [3,4]. By doing so, it leads to the adoption of positive behaviors and the creation of a healthy environment [5]. HEWs are trained and equipped with appropriate supplies to provide basic and essential promotive and preventive services, as well as selected curative services [6].

HEP has been implemented in three settings of varying socioeconomic, cultural, and environmental conditions. These are the agrarian, pastoralist, and urban HEP [7]. The urban HEP was started in 2009 at the national level to address the health crisis and the HIV/AIDS epidemic in urban areas [8]. This HEP ensures health equity by creating demand for essential health services through the provision of health information at a household level and access to services through referrals to health facilities.

UHEP is expected to provide 15 packages. The services are grouped into four main themes: hygiene and environmental sanitation, family health care, prevention and control of communicable and non-communicable diseases, and injury prevention, control, first aid, referral and linkages [9]. Community utilization of the health service is directly affected by their satisfaction with the services they are receiving. Asking the community about how they perceive about the services they have been receiving is an important step towards improving the quality of care, and ensuring the PHC services to meet the community’s needs [10]. Additionally, assessing the community satisfactions was critical to improve health coverage and better delivery of services. Urban health extension workers (UHEWs); are nurses with special training on UHEP designed to strengthen the ability of UHEWs to identify the most at risk populations in the community and provide public health services. They are required to spend 75% of their time conducting outreach activities i.e. conducting house to house activities in the community [11].

The UHEP in Hadiya Zone began in 2009, within few months after the national program have been launched. Nurses were deployed after receiving four month training on UHEP, subsequently who become urban health extension workers (UHEWs) [12]. Previous studies related to HEP were primarily implemented in rural areas. However, more recently programs have been started in urban areas to address urban health problems and there has been an increased need to understand community satisfaction towards UHEP. Therefore, this study was aimed to provide insight into the overall satisfaction of the community towards UHEP and the services provided by UHEWs in Hadiya Zone.

## Methods

A community based cross-sectional study using mixed methods (i.e. quantitative and qualitative methods) were used. The study was conducted from February 1 to March 2, 2014 in Hadiya Zone. Hadiya zone is situated in southern nations and nationalities regional state of Ethiopia located 232 km to south of Addis Ababa. It has 11 administrative districts and according to 2007 national census projection to 2014 it has an estimated total population of 1, 547, 848. From the total population 765,720 (49.47%) were males and 782,128 (50.53%) were females. Around 11% of the population resides in urban areas. A total of 307,497 households were registered in the zone, which results in an average of 4.9 persons per household [13].The purpose of the qualitative method was for triangulation with quantitative part, and to have an in-depth understanding of community perceptions of the UHEP.

### Sample size determination

The sample size was determined by using the single population proportion formula $\mathrm{n}=\frac{{\left({\mathrm{Z}}_{\left(\frac{\upalpha}{2}\right)}\right)}^2\mathrm{P}\ \left(1-\mathrm{P}\right)}{{\mathrm{d}}^2}$ and by considering the following assumptions: 50% of the population is satisfied with UHEP (p), a confidence interval of 95%, and a margin of error (d) of 5%. This yields a sample size of 384. The final sample size was 426 households due to a 10% possible non-response rate. For the qualitative part of the study, four focus group discussions (FGDs) were conducted involving a total of 29 participants. There were 6–8 participants in each group, ranging in age from 24 to 60 years and drawn from four Kebeles (the smallest administrative structure). Two of the FGDs were with model families (selected households from the kebele; receive training and fully implemented the health extension packages as reported by HEW [14]) and two of the FGDs were with village health committee members. This group is selected purposefully, assuming that they have rich information, because they were involved during the implementation of the program.

### Sampling procedure

All of the towns that started implementing a UHEP in the zone were considered in the sampling process for the selection of the study participants. The total sample size was distributed within all towns proportionate to the total number of households found in each town. The final respondents in each town were selected by systematic random sampling. To select sampled houses, a sampling interval was calculated for each town. When more than one eligible respondent was found in a house, a lottery (a classical simple random sampling) method was used to select one respondent. Adult, age greater than 18 years, permanent resident of the towns were considered as a respondent.

### Measurement

Quantitative data were collected by using a structured questionnaire. The questionnaire was developed after a review of documents, guidelines, and manuals related to UHEPs, and various previous studies conducted in rural areas [15-18].

The questionnaires have four parts. The first part is included the socio-demographic characteristics of respondents; the second part is consisted of questions related to the knowledge of respondents about HEWs, which had a “yes” or “no” answer. Questions to assess the attitudes of respondents were included in the third part and rated on a 5-point Likert scale ranging from (1) (strongly disagree) to (5) (strongly agree), which ranges from 4–20 with Cronbach’s alpha = 0.806. The last part included questions related to the perception of the respondents of the HEWs, which was measured with five different aspects of perception: perceived technical competency of HEWs (ten items with Cronbach’s alpha = 0.678), perceived interpersonal relationship with HEWs (five items with Cronbach’s alpha = 0.752), perceived time spent with HEWs (two items with Cronbach’s alpha = 0.835), perceived way of communication (seven items with Cronbach’s alpha = 0.834) and perceived accessibility of service (five items with Cronbach’s alpha = 0.814).

The overall satisfaction of respondents with the health extension program was considered as a dependent variable. Satisfaction of the community was measured using five variables. Each variable was measured on a 5-point Likert scale ranging from (1) (strongly disagree) to (5) (strongly agree), yielding a total score of 5–25 with Cronbach’s alpha = 0.951. Negatively worded questions were reverse scored so that a higher score reflected higher satisfaction with the UHEP.

The questionnaire was developed in English and translated into the local language, then back-translated to English to check for consistency. A pre-test of the questionnaire was performed using 5% of the study sample size and necessary adjustments were done according to pre-test results. The interview was conducted by trained and experienced data collectors. Supervisors oversaw the data collection process. Interview guidelines were used to guide the FGD and one of the investigators moderated the FGD. In addition, a tape recorder was used to record the discussions.

### Data processing and analysis

The quantitative data were entered and analyzed using SPSS version 16. Descriptive statistics and mean scores were used to summarize data, and bivariate analysis was conducted to determine the association of independent variables and community satisfaction. All variables with p-value <0.05 during the bivariate analysis were entered into multivariate linear regression for further analysis. The final model was constructed using stepwise linear regression to identify independent predictors of satisfaction. A P-value < 0.05 was considered statistically significant.

The data from FGD were transcribed verbatim from the tape recorder and transcripts were checked for reliability. The data were analyzed manually by categorizing into different themes and triangulated with the quantitative study.

Ethical approval of the study was obtained from the Addis Ababa University, College of Health sciences ethical review committee. The participants were informed about the purpose of the study and oral consent was obtained from each study participant prior to conducting the interview.

## Results

### Socio-Demographic Characteristics

407 respondents were interviewed using structured questionnaire, yielding a response rate of 95.5%. Of the total respondents, 73.2% were females and 43.7% were aged between 25 and 34 years. 335 (82.3%) respondents were married. Regarding educational status, 32.2% of the respondents were college graduates and 29.5% of them had attended secondary school. With respect to religious affiliation, 65.8% of the respondents were Protestant Christian. Occupationally, 34.2% of the respondents were government employees and 33.7% were housewives. Ethnically, the majority of the respondents (69.8%) were Hadiya (Table **1**).

**Table 1:** **Socio demographic characteristics of the respondents in Hadiya Zone, South Ethiopia, March 2014**

**Background characteristics**		**Frequency**	**Percentage**
**Sex**	Female	298	73.2
	Male	109	26.8
**Age**	18-24	75	18.4
	25-34	178	43.8
	35-44	87	21.4
	45-54	36	8.8
	55+	31	7.6
**Marital status**	Married	335	82.3
	Single	48	11.8
	Divorced	16	3.9
	Widowed	8	2.0
**Educational level**	Illiterate	45	11.0
	Grade 1-8	111	27.3
	Grade 9.12	120	29.5
	College and above	131	32.2
**Religion**	Protestant	268	65.8
	Orthodox	88	21.6
	Muslim	27	6.6
	Catholic	24	5.9
**Occupation**	Gov’t employee	139	34.2
	House wife	137	33.7
	Merchant	59	7.6
	Daily laborer	31	14.5
	Others*	41	10.1

*Farmer, student.

### Respondents’ Knowledge and Attitudes towards the UHEP

The mean score of community knowledge about the UHEP was 4.852 (SD ±1.156) and 278 (68.3%) scored above or equal to the mean knowledge score. Thus, 68.3% of the respondents were well informed about the UHEP.

Similarly, the mean value of community attitude towards UHEP was 15.728 (SD ± 2.814). Two hundred and fifty eight (70.1%) respondents rated their attitude above or equal to the mean value. Therefore, 70% of respondents had a favorable attitude towards the UHEP.

In the qualitative study, the majority of the FGD participants had a positive attitude towards the UHEP. The UHEP addressed health problems such as HIV/AIDS, improper use of the latrine, child health issues, poor household solid waste disposal mechanisms, and poor environmental sanitation. For example, one discussant from the Kebele health committee said:

“…*Truly speaking,since urban health extension professionals started working in our Kebele, we are giving due emphasis for environmental sanitation activities. We have prepared separate solid and liquid waste disposal pit in our household which helped us to prevent ourselves and our family from different health problems”* (A 42-year-old female participant).

Concerning community participation in the planning and implementation of the program, 250 (61.4%) community members did not participate in the planning process. In the qualitative study, the majority of the respondents ascertained that their participation in the planning and implementation of the program helped achieve the desired outcome. Even though community members had good relationships with health extension workers during home visits, the health extension workers did not involve the community in the planning of the program. One discussant said:

*“…I heard about the type of services provided by urban health extension program from health extension workers. I did not attend in any meetings for planning of the service provided by health extension program. If I can participate in the planning, I will contribute what is expected from me in the implementation of the program.”* (46-year- old male discussant).

### Relationship with Urban Health Extension worker

Regarding the relationship of HEWs with the community, 356 (96.7%) of respondents had good relationships with HEWs (Table 2). One of the FGD participants said:

**Table 2:** **Respondents’ experience with urban health extension professionals in Hadiya Zone, South Ethiopia, March 2014**

**Variables**		**Frequency**	**Percentage**
**Participated in planning and implementation of UHEP**	Yes	118	32.1
	No	250	61.4
**Relationship with UHE-Ps**	Excellent	87	23.6
	Very good	145	39.4
	Good	124	33.7
	Poor	11	3.0
	Very poor	1	0.3
**Females are competent to deliver service**	Yes	186	50.5
	No	182	49.5

*“…She (HEW) acts like my child when she comes to my home. In addition, she discusses with me about my personal health problems with respect and in a friendly manner. She sees my problem as hers.”* (48-year-old female discussant).

### Competency of Health Extension worker

186(50.5%) respondents said that HEWs were competent to deliver services. The participants in the qualitative study also supported this idea. They preferred to discuss their personal health issues with females rather than males.

*“…It is easier for me to discuss all my issues with females. If health extension workers are males, I cannot discuss my personal health problems freely. For example if I want to ask them about family planning, I can only talk freely with females.”* (27-year-old female discussant).

### Exposure to Urban Health Extension Packages

368 (90.4%) of the respondents were visited or got advice/service from UHEPs one year prior to the study period. Even though UHEP is designed to provide services in 15 different packages, UHEPs gave more attention to some of the programs. For example, 91.8% and 89.7% of the respondents received services on environmental sanitation and latrine use respectively. Programs such as the prevention of accidents, malaria, TB/leprosy, and non-communicable diseases were given the least attention, even though these are serious health problems in urban areas (Table 3).

**Table 3:** **Community exposure to health extension packages in Hadiya Zone, South Ethiopia, March 2014**

**Health extension packages**	**Frequency**	**Percentage**
**Mental Health**	112	30.4
**Non-communicable disease**	123	33.4
**Malaria**	131	35.6
**Accident Prevention**	132	35.9
**Nutrition**	140	38
**TB and Leprosy**	149	40.5
**Breast feeding**	153	41.6
**Delivery**	154	41.8
**HIV/AIDS**	157	42.7
**Personal Hygiene**	251	68.2
**Antenatal care**	256	69.6
**Family planning**	268	72.8
**Immunization**	305	82.9
**Latrine use**	330	89.7
**Environmental sanitation**	338	91.8

### Perception of the Community on Satisfaction Sub-Scales

Community perception of the services provided by the UHEP was assessed in five key aspects of satisfaction. The mean score was calculated for each sub-scale of satisfaction after which they were summed and converted into percent values for comparison. The highest mean score was found for perceived UHEPs communication with the community (81.59 ± 7.83). The FGD discussants also gave more attention to UHEPs communication during service provision (Table 4).

**Table 4:** **Perception of the community on interaction with HEWs in Hadiya Zone, South Ethiopia, March 2014**

**Variables**	**No of items**	**Mean**	**SD**	**Range of possible score**
**Perceived technical competency**	10	73.46	8.26	20-100
**Perceived interpersonal r/n ship**	5	77.00	7.00	20-100
**Perceived time spent**	2	60.76	6.84	20-100
**Perceived way of communication**	7	81.59	7.83	20-100
**Perceived accessibility**	5	74.81	16.10	20-100

### Overall Community Satisfaction

The mean score of overall community satisfaction with the UHEP was 72.82(SD ±22.09; possible range of responses 20–100). The majority (67.4%) of the respondents had an overall satisfaction score above or equal to the mean value. Thus, 67.4% of the respondents were satisfied with the services provided by the UHEP in the Hadiya Zone.

### Predictors of Overall Community Satisfaction

#### Attitude and Knowledge about the UHEP as Predictors of Satisfaction

The association between respondents’ attitudes and knowledge of the service provided by the UHEP with community satisfaction was analyzed using multiple linear regression through stepwise method. Bivariate analysis showed that respondents’ knowledge and attitudes towards the UHEP were associated with satisfaction of the community.

Multivariate analysis also showed that both attitudes and knowledge towards the UHEP predicted a community’s satisfaction with the UHEP. Accordingly, for a unit increase in the attitude score of respondents, the satisfaction score increased by an average of 3.002 (**ß**; 95%CIs: 02.26, 3.74). Similarly, the satisfaction score of respondents’ increased by an average of 2.302 as the knowledge score increased by one unit (Table 5).

**Table 5:** **Knowledge and attitude of respondents on UHEP as a predictor of satisfaction inHadiya Zone, South Ethiopia March 2014**

**Variable**	**Standardized ß**	**95% CI for ß**	**P- value**
**(Constant)**	13.98	−1.302, 29.27	0.000
**Attitude**	3.002	2.26, 3.74	0.001
**Knowledge**	2.302	0.163, 4.44	0.035

### Communities’ Perception of the UHEP as Predictors of Satisfaction

The association between satisfaction and perception of the community with the services delivered by UHEPs was analyzed by multiple linear regression analysis using a stepwise method to build the model. This model explained 49.7% of the variation in community satisfaction. Bivariate analysis showed that most of the variables were significant except for perceived time spent. Analysis with multivariate linear regression showed that only perceived technical competency, perceived interpersonal relationships, and perceived accessibility of the service were significantly associated with the satisfaction of the community.

Accordingly, as respondents perceived technical competency score increased by one unit, the level of satisfaction increased by an average of 0.425 (**ß**; 95% CIs: 0.16, 0.68). Similarly, for a one-unit increase in respondents’ perceived interpersonal relationship with HEWs, the level of satisfaction increased by an average 0.506(**ß**; 95% CIs: 0.216, 0.797). In addition, for a one-unit increase in respondents’ perception on accessibility of service, the respondents’ satisfaction had an average increase by 0.752 (**ß**; 95% CIs: 0.64, 0.86)(Table 6).

**Table 6:** **Multiple linear regression of the predictors of community satisfaction among respondents of Hadiya Zone, South Ethiopia, March 2014**

**Explanatory variable**	**Standardized ß**	**95% CI for ß**	**P-value**
**Constant**	−53.67	−72.00, −35.33	0.000
**Perceived technical competency**	0.425	0.16, 0.68	0.001
**Perceived interpersonal relation**	0.506	0.21, 0.79	0.001
**Perceived accessibility**	0.752	0.64, 0.86	0.001

## Discussion

This study aimed to assess community satisfaction with the UHEP. The UHEP is a new program and an innovative platform for delivering primary health care services to urban families of Ethiopia [9]. There is a very limited prior study in this program area. In the present study, the mean score of community satisfaction with five of the services provided by the UHEP was 72.82 (SD ± 22.09). Thus, 67.4% of the respondents were satisfied with these services provided by the UHEP. This figure is slightly lower than the study conducted in the Jimma Zone in a rural community, in which 69.9% of the respondents were satisfied with the services provided by rural HEWs [16]. This might be due to the difference in the study area or the status of HEWs and approaches of urban health extension professionals. The rural health extension program was initiated in 2003 [2], about seven years earlier than the UHEP [12].

About half of the respondents think that females are competent to deliver the services of UHEP. This result is consistent with other studies conducted in rural areas [19], and can be explained by the close relationship between respondents and their mothers. The UHEP services addressed maternal and child health issues as parts of the packages and primarily mothers were available at home when services were provided [9].

The objectives of HEP can be achieved if the community is involved in planning and implementing the programs, and therefore; have a voice about their own health and health care [20]. In the present study, however, 61.4% of the respondents did not participate in the planning and implementation of the program. This finding is consistent with a summary of findings presented by an expert review panel [21]. This may be because the majority of the respondents were full-time employees and they may not have had a convenient time to participate in different community meetings and discussions arranged by urban health extension professionals. Besides; irregularity in schedule of meetings, skills of HEWs to engage with community could also be possible reasons.

More than 90% of the respondents had received advice or services from urban health extension professionals in the year prior to the study. This is much higher than that of study conducted in the rural health extension in Wolayita Zone [19]. The differences may be attributable to socio-cultural variations, in that information is more readily available to urban residents than rural residents. Significant proportions of households received information from a newly implemented strategy called the Health Development Army, which is a network of households under the leadership of one household recognized by the urban extension worker to be a model family [8,16]. The leader is expected to gradually influence the group of households with positive attitudes and skills towards healthy behavior [8].

Concerning communities’ relationships with HEWs, the majority of the respondents stated their relation was good, very good, or excellent. This is consistent with the study conducted in Tigray region [22]. This is mainly because most of the users of the service were women and the service was provided by female HEWs.

This study also showed that perceived technical competency, a perceived interpersonal relationship, and perceived accessibility of the services were independent predictors of community satisfaction (P < 0.05). Other studies conducted in rural health extension and PHC services showed that perceived way of communication, perceived respect, and perceived technical skill and competency of HEWs were predictors of satisfaction [16,23-26]. Even though it is difficult to compare the perception of urban respondents with PHC services with that of rural residents, there are some factors that are common to both settings, such as perceived way of communication and perceived technical competency of health care providers.

This study has some limitations which include; the information on the satisfaction may not reflect the actual situation that may be observed in the various seasons of the year which could be addressed via follow-up study. This study was focused demand side point of view (point of households) and supply side view (provider side) was not focused, thus does not indicate the actual utilization of the service. The study was employed using interviewer administrated questioner that might result social desirability bias.

## Conclusion

Both the quantitative and qualitative components of the present study have clearly shown that the majority of the respondents were satisfied with the services provided by the UHEP. The majority of respondents preferred female to deliver UHEP services, and respondents have favorable attitude and good knowledge on UHEP.

Community perceptions of technical competency and interpersonal relationships with urban health extension professionals, as well as the accessibility of the service, were identified as independent predictors of community satisfaction. This study is considered the satisfaction of the community with UHEP services. Further study is needed considering the aspect of provider, the actual utilization and the quality of the service provision.
